# Development and validation of a TaqMan RT-PCR method for identification of mayonnaise spoilage yeast *Pichia kudriavzevii*

**DOI:** 10.1186/s13568-018-0716-y

**Published:** 2018-11-22

**Authors:** M. Y. Syromyatnikov, S. V. Kiryanova, V. N. Popov

**Affiliations:** 1 Department of Genetic, Cytology and Bioengineering, Voronezh State University, 1 Universitetskaya pl., Voronezh, 394018 Russia; 2 Innovative Center “Biruch-NT”, EFKO Group of Companies, Malybykovo, 1 Belaya Vezha Str., Belgorod Region, 309927 Russia

**Keywords:** Mayonnaise spoilage, *Pichia kudriavzevii*, TaqMan, Identification, Mayonnaise screening

## Abstract

Food spoilage and its contamination with yeast and mold is a serious problem of food industry. Despite the high fat content, mayonnaise is an attractive substrate for food spoilage microorganisms. The aim of this study was to develop a method for yeast identification in mayonnaise and to test commercially available mayonnaises for the presence of these contaminating microorganisms. Based on the sequencing of intergenic regions ITS1 and ITS2, we identified a yeast microorganism that causes mayonnaise spoilage. We found that DNA sequences were more than 99% identical to the GenBank DNA sequences from *Pichia kudriavzevii*. We developed a specific to *P. kudriavzevii* TaqMan probe and primers. The reaction conditions were optimized regarding to the components concentration and temperature cycle. The minimum amount of *P. kudriavzevii* DNA that could be detected by developed method was 50 fg. The minimal number of *P. kudriavzevii* cells that could be detected by developed method without pre-enrichment was 50. We tested verified method with DNAs from microorganisms of different taxonomic groups that were obtained from three collections of microorganisms. Finally, we analyzed 20 different brands of mayonnaise from 14 producers and 10 different brands of mayonnaise sauce from seven producers. We determined the Cq parameter that characterizes transition of the fluorescence curve to the logarithmic phase and, therefore, correlates with the extent of sample contamination with *P. kudriavzevii* yeast. *P. kudriavzevii* was detected in six analyzed samples of mayonnaise and one sample of mayonnaise sauce.

## Introduction

Food spoilage and its contamination with pathogenic microorganisms is a serious problem of food industry. Often, contaminating agents are eukaryotic microorganisms, such as yeast and molds. Microbial contamination is very common for the products with a high fat content, e.g., mayonnaises. Mayonnaise is a semi-liquid emulsion of oil, egg yolks, whole eggs, vinegar, and/or lemon juice and some other ingredients (salt, spices, glucose, etc.) with pH 3.6 to 4.6 (Jay et al. 2005). The risk of spoilage is exacerbated by the refusal of food-producing companies to use preservatives. Despite the high fat content, mayonnaise is an attractive substrate for food spoilage microorganisms and even some pathogenic microbes. One of the first studies on mayonnaise contamination was performed by Fabian and Wethington (1950), who identified yeast in mayonnaise samples; other authors have demonstrated later that yeast, e.g., *Saccharomyces bailii*, often contaminates mayonnaise (Kurtzman et al. 1971). The prevalent contaminating bacteria in mayonnaises are halophilic/halotolerant species, such as *Micrococcus* and *Bacillus* (Sagdic et al. 2017).

Among potential sources of microbial contamination are eggs, because egg yolk is one of the main mayonnaise ingredients. Bacteria, such as *Escherichia coli*, *Shigella* spp. and yeasts, such as *Candida albicans* (Lee et al. 2017), and *Coxiella burnetii* (Tatsumi et al. 2006) were found in eggs; for the latter, the identification method using real-time PCR has been developed (Sadamasu et al. 2006). In another study, *Candida* sp. was found in 231 out of 380 mayonnaise samples tested (Musgrove et al. 2008). Other common eukaryotic egg-contaminating microorganisms are molds, mostly from the genera *Mucor*, *Penicillium*, *Hormodendron*, and *Cladosporium*; *Penicillium* and *Cladosporium* being the most frequent causes for fungal spoilage of eggs (Jay et al. 2005). Representatives of other genera, such as *Cryptococcus, Hansenula, Hyphopichia, Metschnikowia, Rhodotorula, Sporobolomyces*, and *Torulaspora* have been identified in eggs as well. The most dangerous mayonnaise-contaminating microorganism is *Salmonella*. In Rio Grande do Sul (Brazil), homemade mayonnaise accounts for 17% cases of salmonellosis (Capalonga et al. 2014). Changes in pH and temperature can control the growth of *Salmonella enteritidis* (Keerthirathne et al. 2016), and the model describing the growth of this microorganism in mayonnaise at different temperatures has been proposed (Elias et al. 2016).

Scientists have also been searching for biologically active compounds capable of inhibiting microbial growth in mayonnaise. For example, chitosan was found to suppress the growth of *Lactobacillus*, but not of the yeast *Saccharomyces bailii* (Oh et al. 2001).

The common method for identification of microorganisms in mayonnaise is microbial plating on a nutrient medium; however, molecular genetic methods have also gained a wide recognition, because these methods are considerably less time-consuming. Thus, real-time PCR method was suggested for *Shigella* sp. detection (Villalobo and Torres 1998) and successfully used for *Salmonella enterica* identification in mayonnaise (Almeida et al. 2013). Another real-time PCR-based system (MicroSEQ^®^ L. monocytogenes Detection Kit) was used for *Listeria monocytigenes* detection in mayonnaise-containing food products (Tebbs et al. 2011). A method for differentiation of contaminating yeasts have been developed based on the high-resolution melting analysis (Erdem et al. 2016); however, this method did not provide rapid and precise identification of *Pichia* spp.

The aim of this study was to develop a method for yeast identification in mayonnaise and to test commercially available mayonnaises for the presence of these contaminating microorganisms.

## Materials and methods

### Samples

Commercially available mayonnaises and mayonnaise sauces of different brands available in Russia market were bought for testing and stored at + 4 °C in sealed packaging before analysis.

### Microbiological analysis

To test for microbial contamination, mayonnaise samples (1 ml) were diluted two times and plated on the medium containing 40 g/l glucose, 10 g/l peptone, and 15 g/l agar. All medium components were dissolved in hot water, and pH was adjusted so that pH after sterilization was 6.5 at 25 °C. Chloramphenicol was added at a final concentration of 50 mg/l.

To enrich yeast cells, mayonnaise samples (1 ml) were enriched by incubating for 19 h in 30 ml of Sabouraud broth containing 20 g/l peptone and 40 g/l glucose, and then centrifuged for 5 min at 10,000*g*. The resulting pellet was used for DNA isolation.

### Molecular identification of yeasts

DNA from the grown colonies was isolated using commercially available PROBA-GS kit (DNA Technology, Russia). Polymerase chain reaction was performed with an Eppendorf MasterCycler Personal cycler. Each PCR reaction mixture contained 2.5 µl of 10× reaction buffer, 1 µl of 10 mM dNTPs, 1 µl of 10 µM forward primer, 1 µl of 10 µM reverse primer, 3 µl of 25 mM Mg^2+^, 1 µg of template DNA, 2.5 units of thermostable *Taq* DNA polymerase (Evrogen, Russia), and deionized water (up to 25 μl). PCR regime included initial denaturation at 94 °C for 5 min; 35 cycles of denaturation at 94 °C for 30 s, annealing at 54 °C for 30 s, elongation at 72 °C for 45 s; final elongation at 72 °C for 10 min. Fungal-specific primers for molecular identification were: direct ITS1 primer—5′-TCCGTAGGTGAACCTGCGG; reverse ITS4 primer—5′-TCCTCCGCTTATTGATATGC (White et al. 1990).

PCR products were stained with ethidium bromide and visualized at 312 nm with a TCO-20LM transilluminator after electrophoresis in 2% agarose gel.

RCR products were purified from the gel with a Cleanup Standard kit (Evrogen, Russia) and sequenced with an Applied Biosystems 3500 automated sequencer using a BigDye Terminator v3.1 Cycle Sequencing Kit and ITS1/ITS4 primers.

### Development TaqMan RT-PCR

Nucleotide sequence alignment was carried out in MEGA6 tool (Tamura et al. 2013). Primer/probe sets for identification of *P. kudriavzevii* were designed according to the following criteria: primer length between 18 and 30 bp; no distinctive hairpin structures and dimers; GC content from 20 to 80%; minimal G/C content at the 3′ end of the primers; minimum identical nucleotides together in TaqMan probes; no G at the probe 5′-end; PCR-product size from 80 to 250 bp; annealing temperature of the probe at least 4 °C above annealing temperature of the primers.

Real-time PCR was carried out in a Bio-Rad CFX96 Real-time PCR Detection System (Bio-Rad, USA) using commercially available qPCRmix-HS mixture (Eurogen, Russia) according to the following protocol: initial denaturation at 94 °C for 5 min; 37 cycles of denaturation at 94 °C for 30 s, annealing at 54 °C for 30 s, elongation at 72 °C for 30 s.

## Results

### Bioinformatics analysis and primer/probe design

To identify contaminating yeast species, we selected eight commercially available mayonnaise samples and plated them on agar plates. After 3-day growth, yeast-resembling colonies were isolated for further molecular biological studies. Yeast presence was found in four samples of mayonnaise. After DNA isolation we sequenced the DNA fragments that contained 5.8S rRNA gene and two internally transcribed spacers, ITS1 and ITS2 and compared them to nucleotide sequences from the GenBank database. We found out that all sequences were more than 99% identical to the DNA sequences from *P. kudriavzevii*. The sequenced fragments were also analyzed using the data from the Boldsystem (http://www.boldsystems.org) that confirmed that the identified microorganism was *P. kudriavzevii*. The obtained sequences were deposited in the Genbank database under the corresponding numbers: MH626402.1, MH626403.1, MH626404.1, MH626405.1, MH626406.1, MH626407.1, MH626408.1 and MH626409.1.

We analyzed over 180 registered in GenBank DNA sequences of 5.8S, 18S and 28S ribosomal RNA genes and internally transcribed spacers ITS1 and ITS2 for *P. kudriavzevii* and synonymic species (*Issatchenkia orientalis*, *Candida krusei*) to identify specific sequences. The primer/probe sets for identification of *P. kudriavzevii* were suggested (Table 1).

**Table 1:** Suggested primers and TaqMan probes for identification of *P. kudriavzevii*

Primer/probe set	Nucleotide sequences	PCR product length, b.p.
1	Forward primer GAATTGCAGCCATCGTGAATC TaqMan probe FAM-TCGTTTCCATCTTGCGCGTGC-BHQ1 Reverse primer CTCCGACGCTCTTTACACG	149
2^a^	Forward primer GCGAAATGCGATACCTAGTG TaqMan probe FAM-TGCAGCCATCGTGAATCATCGAGT-BHQ1 Reverse primer GATGGAAACGACGCTCAAA	114
3	Forward primer GCGGACGACGTGTAAAG TaqMan Probe FAM-GAGCGAGTGTTGCGAGACAACAA-BHQ1 Reverse primer CGGGTATTCCTACCTGATTTG	156
4	Forward primer GAGCGTCGTTTCCATCTT TaqMan probe FAM-CAGCTCCGACGCTCTTTACACGTC-BHQ1 Reverse primer CAGCTTCGCTCCCTTTC	102
5	Forward primer GATCTCTTGGTTCTCGCATC TaqMan probe FAM-CACACTAGGTATCGCATTTCGCTGC-BHQ1 Reverse primer GCGTTCAAGAACTCGATGA	92
6	Forward primer GCAGCCATCGTGAATCAT TaqMan probe FAM-TTTGAGCGTCGTTTCCATCTTGCG-BHQ1 Reverse primer AGCTCCGACGCTCTTTA	146

^a^ The most optimal primer/probe sets for identification of *P. kudriavzevii*

**Fig. 1:**
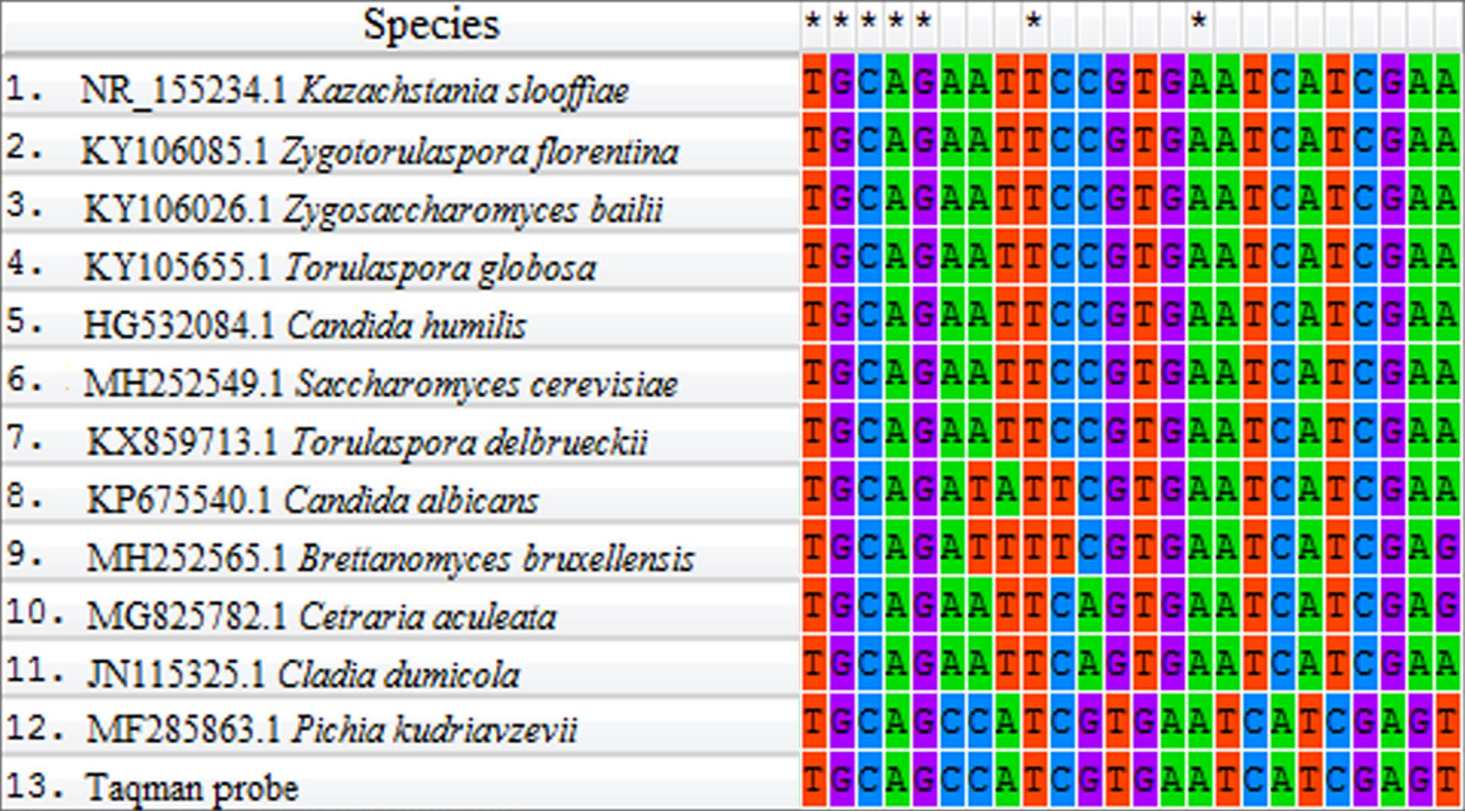
Homologous sequences of the *P. kudriavzevii* DNA and DNA of other closest eukaryotic microorganisms

More rigorous analysis of the designed primer/probe sets revealed possible heterodimer (8 nucleotides) formation between the TaqMan probe and direct primer and between the TaqMan probe and reverse primer in the primer/probe sets 4 and 6, respectively, so they were omitted from further study. The remaining four primer/probe sets were synthesized and verified in real-time PCR with yeast DNA. No TaqMan probe hybridization was observed for the sets 1 and 3; out of sets 2 and 4, TaqMan probe from set 2 hybridized more strongly and at earlier PCR cycles, so this primer/probe set was used in further studies. Specificity to *P. kudriavzevii* DNA mostly provides TaqMan probe and a reverse primer from set 2. The site for TaqMan probe hybridization is specific for this yeast. Homologous sites of the TaqMan probe hybridization to DNA of the closest eukaryotic microorganisms registered in the GenBank are shown in Fig. 1.

**Fig. 2:**
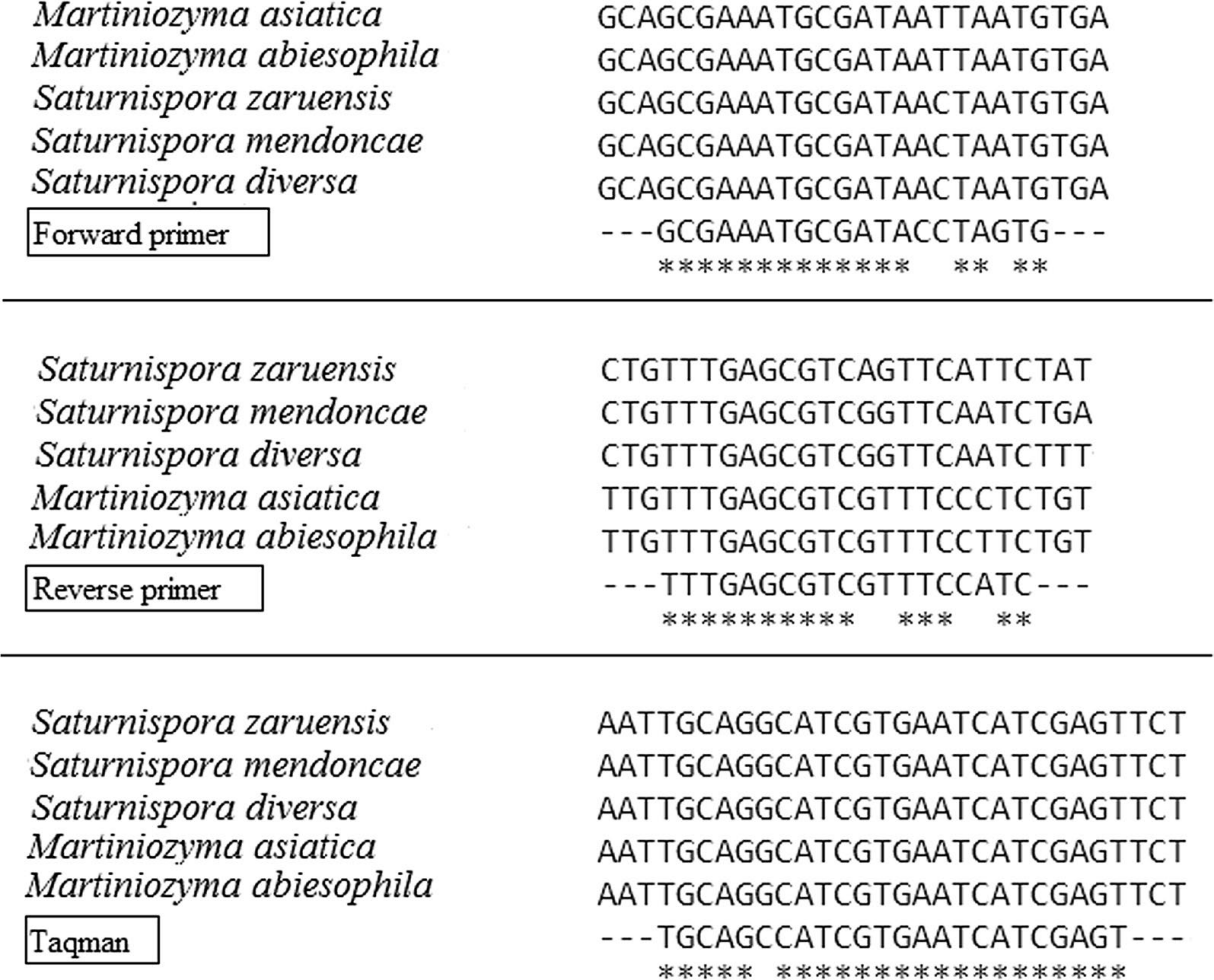
Sites for primers and TaqMan probe hybridization of *P. kudriavzevii* DNA and *Martiniozyma* sp. and *Saturnispora* sp. DNA

The site for attaching the TaqMan probe to DNA was highly specific to this species of yeast. There was no variability of this site inside the *P. kudriavzevii* species. However, it was found that there is still a small probability of a positive TaqMan reaction with DNA from yeasts that phylogenetically most close to *Pichia* sp.—*Martiniozyma* sp. and *Saturnispora* sp. (Fig. 2).

Based on the PCR product expected size and the primer annealing temperature, the PCR regime used was as follows: 94 °C for 4 min followed by 37 cycles of 94 °C for 30 s, 50–58 °C for 30 s, 72 °C for 30 s. We ran the reaction at five different primer annealing temperatures: 50, 52, 54, 56, and 58 °C. At all the temperatures tested, the fluorescence curve reached the logarithmic phase at the same cycle number, although the total levels of fluorescence were higher at 50, 52, and 54 °C. Since higher primer annealing temperatures increase primer specificity, 54 °C was chosen as the optimal primer annealing temperature in our experiments. We also tested the effect of TaqMan probe concentration (100, 200, and 500 nM) on the reaction parameters and found that amplification using 200 nM TaqMan probe produced more stable and pronounced amplification curves; the total level of fluorescence with 200 nM TaqMan probe was higher than when using 100 nM TaqMan probe. The optimal number of cycles was 37, because in such a number of cycles fluorescence was not observed in samples without *P. kudriavzevii* DNA.

### Estimation of method sensitivity

**Fig. 3:**
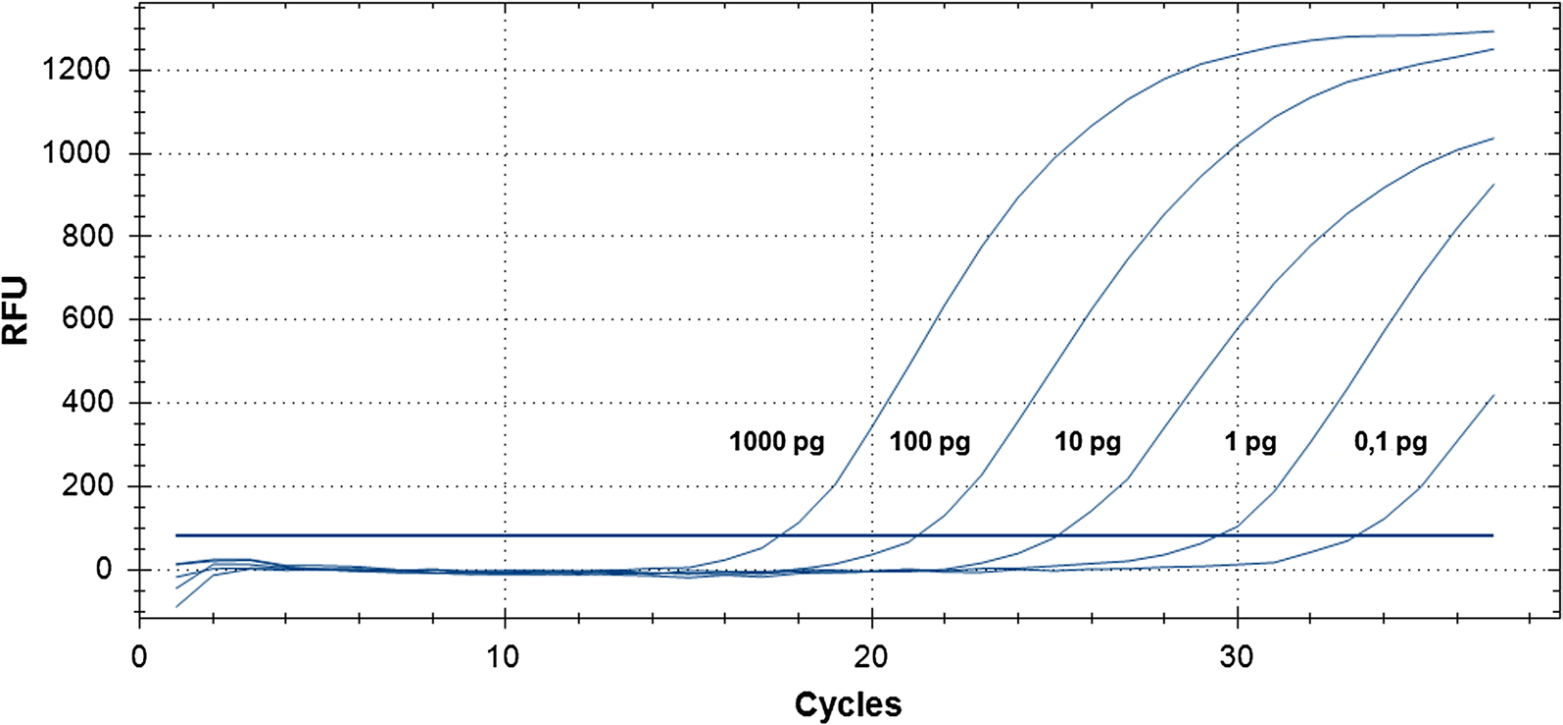
The influence of different amount of *P. kudriavzevii* genomic DNA per reaction and Cq parameter

**Fig. 4:**
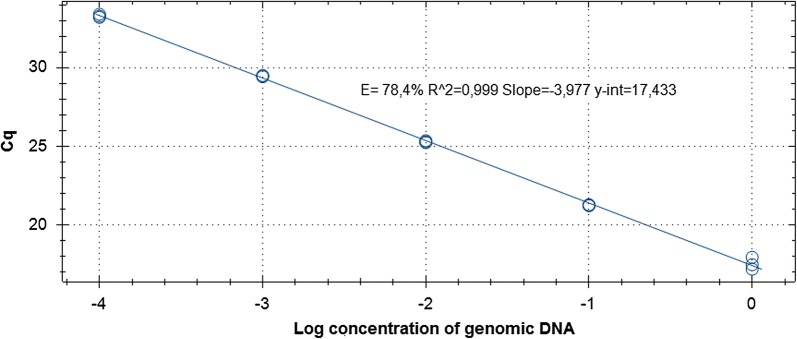
The dependence of Cq parameter on concentration of genomic DNA of *P. kudriavzevii*

DNA was isolated from *P. kudriavzevii* culture and diluted to 1 ng/μl, diluted 1: 10, 1: 100, 1:1000, and 1: 10,000, and used as a template in real-time PCR with the developed primer/probe set (1 μl per reaction tube), see Fig. 3. The linear dependence of the Cq parameter on the concentration of the genomic DNA of *P. kudriavzevii* was observed (Fig. 4), which means that the reaction is specific only to the target DNA without the formation of non-specific products.

The minimum amount of *Pichia* sp. DNA that could be detected by our method was 50 fg. To estimate the minimal number of live cells sufficient for *P. kudriavzevii* detection, total DNA was isolated from 10, 50, 100, 1000, and 10,000 cells of *P. kudriavzevii* and used as a template in the same PCR. The minimal number of yeast that could be detected by our method without pre-enrichment was 50 cells.

### Verification of the specificity of the developed primer/probe set for *Pichia kudriavzevii*

To verify the specificity of the developed primer/probe, we tested it in PCR with DNAs from microorganisms of different taxonomic groups that were obtained from the Russian Collection of Microorganisms (VKM) at the Skryabin Institute of Microbial Biochemistry and Physiology (Pushchino, Russia), State collection of microorganisms that cause dangerous, particularly dangerous, including zooanthroponotic animals diseases (VKOM) at the Federal Research Center for Virology and Microbiology (Pokrov, Russia) and Russian collection of industrial microorganisms (VKMP) State Research Institute of Genetics and Selection of Industrial Microorganisms (Moscow, Russia) (Table 2).

**Table 2:** Verification of the specificity of the developed method with microorganisms from different taxonomic groups

Organism	Collection	Collection number or strain	Reaction with TaqMan
Mold and yeast			
*Alternaria alternata*	VKPM	F300	–
*Aspergillus flavus*	VKPM	F1271	–
*Aspergillus carbonarius*	VKPM	F301	–
*Chaetomium globosum*	VKPM	F323	–
*Fusarium verticillioides*	VKPM	F206	–
*Geotrichum candidum*	VKPM	F220	–
*Penicillium funiculosum*	VKPM	F977	–
*Penicillium pinophilum*	VKPM	F896	–
*Phanerochaete chrysosporium*	VKPM	F615	–
*Phlebia albida*	VKPM	F507	–
*Pleurotus ostreatus*	VKPM	F276	–
*Trichoderma reesei*	VKPM	F184	–
*Trichoderma viride*	VKPM	F179	–
*Brettanomyces custersianus*	VKM	Y-1419	–
*Candida pseudotropicalis*	VKPM	Y190	–
*Candida tropicalis*	VKPM	Y143	–
*Dekkera anomala*	VKM	Y-19	–
*Pichia fermentans*	VKM	Y-244	+
*Pichia kudriavzevii*	VKM	Y-184	+
*Pichia membranifaciens*	VKM	Y-248	+
*Saccharomyces cerevisiae*	VKPM	Y2396	–
*Yarrowia lipolytica*	VKPM	Y34	–
*Zygosaccharomyces rouxii*	VKM	Y-866	–
Bacteria			
*Bacillus subtilis*	VKPM	B5250	–
*Cellulomonas flavigena*	VKPM	B2560	–
*Corynebacterium glutamicum*	VKPM	B1003	–
*Lactobacillus plantarum*	VKPM	B3487	–
*Pseudomonas fluorescens*	VKM	B-894	–
*Streptomyces fulvoviridis*	VKPM	AC-605	–
*Listeria monocytogenes*	VKOM	766	–
*Staphylococcus aureus*	VKOM	209-P	–
*Pseudomonas aeruginosa*	VKOM	273	–
*Bacillus cereus*	VKOM	96	–
*Escherichia coli*	VKOM	К12	–
*Salmonella enteritidis*	VKOM	5765	–
*Listeria ivanovii*	VKOM	7842	–
*Micrococcus luteus*	VKOM	4698 ATCC	–
*Rhodococcus equi*	VKOM	5869	–

PCR proceeded only with microorganisms from the *Pichia* genus. No increase in fluorescence was observed for other yeast, molds and bacteria. We also contaminated 23 samples of preliminary tested microbe-free mayonnaise with *P. kudriavzevii*. After these samples were enriched, DNA was isolated and used in the real-time PCR with the developed primer/probe set. In all 23 cases, the reaction was positive, i.e., the reproducibility of the method was 100%.

### Testing commercially available mayonnaises and mayonnaise sauces for microbial contamination

We analyzed 20 different brands of mayonnaise from 14 producers and 10 samples of mayonnaise sauce from seven different brands. We determined the Cq parameter that characterizes transition of the fluorescence curve to the logarithmic phase and, therefore, correlates with the extent of sample contamination with *P. kudriavzevii* yeast.

The increase in fluorescence was observed in six analyzed samples of mayonnaise: sample 1 (Cq = 20,13), sample 13 (Cq = 31,70), sample 14 (Cq = 28,61), sample 15 (Cq = 30,40), sample 17 (Cq = 33,95) and sample 17 (Cq = 34,50). 1. The increase in fluorescence was observed in sample 1 of mayonnaise sauce (Cq = 34,86). The Cq values for the “positive” samples differed, thereby indicating different extents of contamination with *P. kudriavzevii*.

## Discussion

Detection and quantitative estimation of yeast and mold contamination is a sanitary requirement for most food products, although contaminating microorganisms are not usually identified to genera or species. Different yeast and molds might develop differently in different food products. The yeast *P. kudriavzevii*, that were identified by us in mayonnaise samples, tolerate high fat content and low pH and were detected in Chinese vinegar (Li et al. 2014). *P. fermentas* were found in freshly cut apples (Graça et al. 2015); *P. anomala* were identified in maize silage that it characterized by low pH values (Santos et al. 2017). *Pichia* yeast has been also found in other products: *P. guilliermondii* (Sangorrin et al. 2013), *P. membranifaciens*, and *P. manshurica* contaminate wines; *P. fermentans* and *P. kudriavzevii* were found in cheeses (Gonçalves Dos Santos et al. 2017). Also, yeasts of these taxonomic groups were identified in products with high fat content (Arroyo-Lopez et al. 2012). Therefore, early detection of *P. kudriavzevii* yeast will ensure removal of contaminated products from realization and eliminate the use of infected ingredients in the production of mayonnaise and mayonnaise sauces.

In this study, we developed a primer/probe set to a region of *Pichia* genome that included internally transcribed spacer ITS1, 5.8S rRNA gene and internally transcribed spacer ITS2. This region was chosen because the information on its nucleotide sequence in many organisms (including *Pichia* yeast) is widely available in sequence databases, such as GenBank. In addition, the genomic DNA of eukaryotic microorganisms contains this region in several copies. This increases the sensitivity of the method. Using this information, we were able to identify taxon-characteristic conserved nucleotide sequences and to develop a corresponding probe/primer set for *P. kudriavzevii* identification. The primer/probe set used in this study was highly specific and did not anneal to the DNA from other than *Pichia* microorganisms. The sensitivity of our method without pre-enrichment was 50 yeast cells.

We have found that primers and the TaqMan probe can react with DNA of other species from the *Pichia* genus. Given the purpose for which the identification system was developed (detection of mayonnaise spoilage yeast), this fact does not have any negative impact. For the same reason, the hypothetical interaction of the developed TaqMan probe with DNA of *Martiniozyma* sp. and *Saturnispora* sp. also will not have a negative effect, because these yeasts are very close to *Pichia* sp. (Kurtzman et al. 2008; Kurtzman 2015). We couldn’t check experimentally whether there is a TaqMan probe interaction with DNA of these microorganisms because we did not have verified collection strains of the *Martiniozyma* sp. and *Saturnispora* sp.

The developed method for rapid identification of *Pichia* yeast using real-time PCR reduces the time required for analysis from 5 days to 24 h, which allows rapid detection of contamination source and timed removal of the contaminated produce.

Molecular genetic identification of microorganisms in food products with high fat content (such as mayonnaise) is often impeded by the difficulties in the DNA isolation. In the procedure developed by us, yeast cells were concentrated by centrifugation after preliminary enrichment of the culture. By doing this, we solved both methodical problems, i.e., we increased the method sensitivity by enriching the microorganism culture and removed fats that interfered with DNA isolation.

The developed procedure is rapid and simple and could be carried out in any laboratory equipped with a real-time PCR system. It could also be used for testing other food products.

Screening of commercially available mayonnaises and mayonnaise sauces demonstrated that 6 out of 20 mayonnaises and 1 out of 10 mayonnaise sauces were contaminated with *Pichia* yeast.

As follows from the high Cq value, one of the mayonnaise samples displayed an extremely high abundance of *Pichia* sp. cells; two other samples were moderately contaminated, and the rest of the samples exhibited low extent of yeast contamination.

Note that the positive count in mayonnaises was higher than in mayonnaise sauces due, probably, to the fact that mayonnaise sauces contain preservatives, while mayonnaises are often positioned as “organic” products free of chemical additives, such as preservatives or aromatic compounds.

In conclusion, we identified yeasts that able to contaminate mayonnaise. Based on the sequencing of intergenic regions ITS1 and ITS2, we shown that spoilage mayonnaise yeasts are *P. kudriavzevii*. We developed a specific to *P. kudriavzevii* TaqMan probe and primers. The reaction was optimized for the components concentration and temperature cycle. The minimal number of yeast cells that could be detected by our method without pre-enrichment was 50 cells. Screening of commercially available mayonnaises and mayonnaise sauces demonstrated that 6 out of 20 mayonnaises and 1 out of 10 mayonnaise sauces were contaminated with *Pichia* yeast. The developed procedure is rapid and simple and could be carried out in any laboratory equipped with a real-time PCR system.

## Abbreviations

DNA: deoxyribonucleic acid
RNA: ribonucleic acid
PCR: polymerase chain reaction
ITS: internal transcribed spacer
